# Strongyloides stercoralis Presenting as Duodenitis and Colitis: A Case Report

**DOI:** 10.7759/cureus.112151

**Published:** 2026-07-06

**Authors:** Nabiha Sohail, Saira Butt, Nikolay Popnikolov

**Affiliations:** 1 Internal Medicine, Cavalry Hospital, Lahore, PAK; 2 Internal Medicine and Infectious Diseases, Indiana University School of Medicine, Indianapolis, USA; 3 Laboratory Medicine and Pathology, Indiana University School of Medicine, Indianapolis, USA

**Keywords:** aids, hiv, ivermectin, strongyloides, strongyloidiasis

## Abstract

We report a case of *Strongyloides stercoralis* infection in an Ethiopian female refugee with advanced, untreated HIV who presented with weight loss, dysphagia, gastrointestinal bleeding, and pancytopenia. Duodenal and colonic biopsies confirmed the diagnosis, and the patient was successfully treated with oral ivermectin and initiation of antiretroviral therapy (ART), with full symptom resolution at follow-up. This case underscores the importance of maintaining a high clinical suspicion for strongyloidiasis in immunocompromised refugees from endemic regions, even when classic markers such as eosinophilia are absent.

## Introduction

The parasitic nematode *Strongyloides stercoralis* is widely endemic to tropical, subtropical, and temperate regions of the world, as well as portions of the southeastern United States [1]. Unlike most other helminths, *S. stercoralis* possesses a unique capacity for autoinfection, allowing it to persist indefinitely within the human host for decades without further environmental exposure [1,2]. While chronic strongyloidiasis remains largely asymptomatic or mimics benign gastrointestinal disorders like acid reflux or irritable bowel syndrome, severe clinical presentations can erupt during states of compromised cell-mediated immunity [1].

The prevalence of *Strongyloides* infection is estimated to be approximately 5% among people living with human immunodeficiency virus (HIV) [3]. Although advanced HIV/AIDS carries a less significant risk for accelerated hyperinfection or systemic dissemination compared to co-infection with human T-lymphotropic virus type 1 (HTLV-1) or high-dose corticosteroid treatment, severe localized gastrointestinal disease remains a critical concern [1,4]. Here, we present the case of a 52-year-old Ethiopian female refugee living with advanced HIV who presented with weight loss and acute lower gastrointestinal bleeding, ultimately diagnosed with concurrent *Strongyloides* duodenitis and colitis.

## Case presentation

A 52-year-old Ethiopian female refugee residing in the United States, living with HIV/AIDS, presented to the emergency department. She reported a one-month history of progressive weight loss, generalized weakness, dysphagia, and abdominal pain, acutely exacerbated by dark, tarry stools (melena) over the preceding three days. She was not taking antiretroviral therapy (ART).

On admission, the patient appeared cachectic, but her physical examination revealed no localizing symptoms. Vital signs were notable for a temperature of 102°F. Laboratory investigations revealed profound immunosuppression, with a CD4 T-lymphocyte count of 35 cells/µL and an HIV viral load exceeding 2 million copies/mL. Initial blood counts demonstrated pancytopenia and no peripheral eosinophilia, with a white blood cell (WBC) count of 1.8 x 10³ cells/µL (normal range 4.5 x 10³ to 11 x 10³ cells/µL). The WBC differential included 58% neutrophils (normal range 55% to 70%), 32% lymphocytes (normal range 20% to 40%), and 4% eosinophils (normal range 1% to 4%). She had a hemoglobin level of 5.5 g/dL (normal range 12.1 to 15.1 g/dL), indicating severe anemia, and a platelet count of 41 x 10³ cells/µL (normal range of 150 x 10³ cells/µL to 450 x 10³ cells/µL). A chest X-ray and a contrast-enhanced computed tomography (CT) scan of the abdomen were unremarkable, showing no signs of pulmonary involvement or gross bowel pathology.

**Figure 1:**
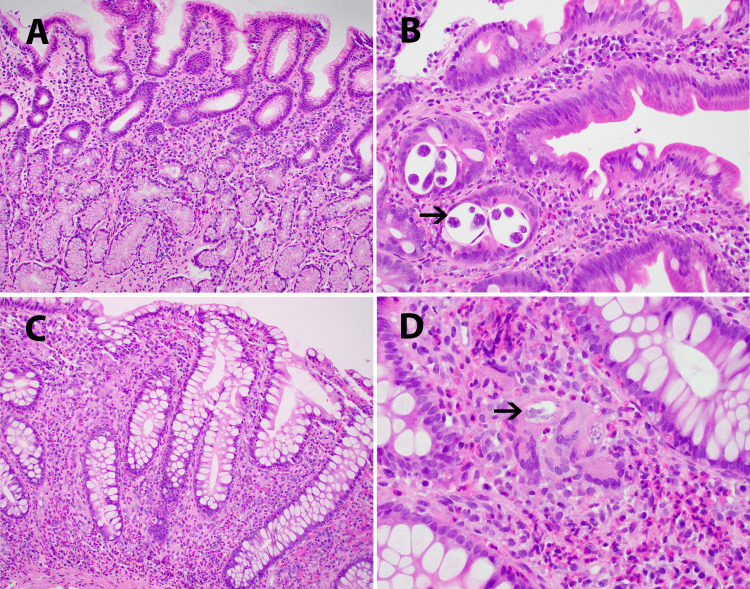
Histopathological features of gastrointestinal Strongyloides infection A: gastric antral mucosa with moderate chronic inactive gastritis and increased eosinophils (x 200); B: duodenal mucosa with *Strongyloides* worms embedded in the crypts (arrow) and associated eosinophil-rich inflammation (x 400); C: colitis with increased eosinophils and focal architectural distortion (x 200); D: focus of worms (arrow) with foreign body reaction in the colonic lamina propria (x 600).

Given her presentation with melena and severe anemia, esophagogastroduodenoscopy with duodenal and gastric biopsies and colonoscopy with colon biopsy were performed. Histopathological evaluation of the tissue biopsies provided the definitive diagnosis. Endoscopic biopsy of the duodenal mucosa revealed active duodenitis characterized by an accumulation of mature *Strongyloides* worms embedded directly within the mucosal crypts, surrounded by an eosinophil-rich inflammatory infiltrate (Figure 1). Colonic biopsies verified active colitis, with marked eosinophilic inflammation, focal architectural distortion, and clusters of worms lodged within the lamina propria, accompanied by a foreign body reaction (Figure 1). No larvae were identified outside the gastrointestinal tract, ruling out hyperinfection or disseminated strongyloidiasis.

The patient was treated with oral ivermectin at a dose of 200 mcg/kg/day for two consecutive days. Concurrently, her clinical condition was stabilized with supportive care, and she was successfully initiated on an appropriate ART regimen (bictegravir, emtricitabine, and tenofovir alafenamide). A repeat two-day course of ivermectin was administered two weeks later to eradicate any newly matured worms from the autoinfection cycle. At her subsequent outpatient clinic follow-up four weeks post the last dose of ivermectin and initiation of ART, the patient demonstrated complete resolution of her gastrointestinal symptoms, gained weight, and tolerated her ART well. Her WBC count was 4 x 10³ cells/µL with 2% eosinophils, a hemoglobin level of 11 g/dL, and a platelet count of 95 x 10³ cells/µL.

## Discussion

The pathogenesis of *S. stercoralis* begins when infective filariform larvae penetrate intact human skin, travel via the venous circulation to the lungs, ascend the tracheobronchial tree, are swallowed, and settle in the small intestine to mature into parthenogenetic adult females [1,5]. These females lay eggs that quickly hatch into non-infective rhabditiform larvae [2]. Instead of exiting via feces, some rhabditiform larvae prematurely molt into infective filariform larvae within the gastrointestinal lumen, boring through the intestinal mucosa or perianal skin to re-enter the systemic circulation [1,2]. This internal auto-loop allows the parasite to persist inside a single host for decades without re-exposure to an external environmental source [2].

While chronic infections are frequently asymptomatic or oligosymptomatic, presenting with transient, highly pruritic linear skin tracks known as larva currens, intermittent epigastric distress, or mild respiratory irritation [1,6], to severe clinical scenarios when host cell-mediated immunity is compromised [1]. Systemic corticosteroids, malignancy, or co-infection with HTLV-1 can accelerate the autoinfection cycle unchecked [1,4]. This triggers hyperinfection syndrome, an explosion of parasite burden confined to the typical gastrointestinal and pulmonary migration pathways, which can rapidly progress to disseminated strongyloidiasis, in which larvae invade atypical organs such as the central nervous system, kidneys, and liver [1,2]. Disseminated larvae carry enteric bacteria across the disrupted intestinal wall, frequently leading to fatal secondary polymicrobial or Gram-negative bacteremia, sepsis, and acute respiratory distress syndrome, carrying mortality rates exceeding 70% to 80% [1,2].

Diagnosis remains inherently challenging because larval shedding in chronic infections is low and highly irregular, dropping the sensitivity of a single direct stool microscopy smear to roughly 21% [7]. Sensitivity improves markedly with specialized, repetitive parasitological techniques such as the Baermann concentration method (~72%) and nutrient agar plate culture (~89%) [7,8]. While serological assays (e.g., enzyme-linked immunosorbent assay (ELISA)) offer high sensitivity and high negative predictive value, making them excellent screening tools, they suffer from cross-reactivity with other tissue helminths and cannot reliably distinguish past, cleared exposure from an active infection [5,7]. Furthermore, peripheral eosinophilia has proven to be a remarkably poor predictor of *Strongyloides* infection in refugee populations [9]. Molecular approaches like polymerase chain reaction (PCR) offer high specificity, and advanced techniques such as metagenomic next-generation sequencing (mNGS) are proving invaluable for rapid detection in severe, disseminated cases, using bronchoalveolar lavage fluid or blood [10]. In this case, because diagnostic stool methods can be elusive, the diagnosis was definitively established by direct histopathology of endoscopic biopsies, which revealed the worms residing within the mucosal crypts and the colonic lamina propria.

The primary treatment of choice for stable, uncomplicated strongyloidiasis is oral ivermectin, which achieves a near 98% to 100% cure rate when administered as a short, two-day regimen [5,8]. Albendazole serves as an alternative secondary option but demonstrates significantly lower efficacy than ivermectin [5,10]. Conversely, in severe hyperinfection syndrome or disseminated states, treatment necessitates prolonged, multiple-dose therapy or combination regimens until all clinical symptoms clear and successive fluid evaluations yield no larval presence [2,11]. Because ivermectin does not clear migrating larvae or eggs, administering a second two-day course two weeks later remains standard practice to target larvae that have matured since the initial intervention.

## Conclusions

This case underscores that *Strongyloides stercoralis* must be considered in the differential diagnosis of gastrointestinal bleeding in immunocompromised refugees from endemic regions. Due to the unreliable nature of peripheral eosinophilia and routine stool microscopy in advanced AIDS, upper and lower endoscopic tissue biopsies serve as an essential diagnostic path. Early identification and a timely two-dose regimen of ivermectin are curative and critical to preventing potential progression to fatal hyperinfection.
